# Three-Dimensional Genome Organization in Breast and Gynecological Cancers: How Chromatin Folding Influences Tumorigenic Transcriptional Programs

**DOI:** 10.3390/cells11010075

**Published:** 2021-12-28

**Authors:** Stephanie I. Nuñez-Olvera, Jonathan Puente-Rivera, Rosalio Ramos-Payán, Carlos Pérez-Plasencia, Yarely M. Salinas-Vera, Lorena Aguilar-Arnal, César López-Camarillo

**Affiliations:** 1 Departamento de Biología Celular y Fisiología, Instituto de Investigaciones Biomédicas, Universidad Nacional Autónoma de México, Mexico City 04510, Mexico; iraiz.nunez@iibiomedicas.unam.mx; 2 Posgrado en Ciencias Genómicas, Universidad Autónoma de la Ciudad de México, Mexico City 03100, Mexico; jo_puenter@hotmail.com; 3 Facultad de Ciencias Químico Biológicas, Universidad Autónoma de Sinaloa, Culiacan City 80030, Mexico; rosaliorp@uas.edu.mx; 4 Laboratorio de Genómica, Instituto Nacional de Cancerologia, Mexico City 14080, Mexico; carlospplas@gmail.com; 5 Departamento de Bioquímica, Centro de Investigación y Estudios Avanzados, Mexico City 07360, Mexico; yarely.salinas@cinvestav.mx

**Keywords:** 3D genome, cis-regulatory elements, topology-associated domains, CTCF, breast cancer, gynecological cancers, epigenetic regulation

## Abstract

A growing body of research on the transcriptome and cancer genome has demonstrated that many gynecological tumor-specific gene mutations are located in cis-regulatory elements. Through chromosomal looping, cis-regulatory elements interact which each other to control gene expression by bringing distant regulatory elements, such as enhancers and insulators, into close proximity with promoters. It is well known that chromatin connections may be disrupted in cancer cells, promoting transcriptional dysregulation and the expression of abnormal tumor suppressor genes and oncogenes. In this review, we examine the roles of alterations in 3D chromatin interactions. This includes changes in CTCF protein function, cancer-risk single nucleotide polymorphisms, viral integration, and hormonal response as part of the mechanisms that lead to the acquisition of enhancers or super-enhancers. The translocation of existing enhancers, as well as enhancer loss or acquisition of insulator elements that interact with gene promoters, is also revised. Remarkably, similar processes that modify 3D chromatin contacts in gene promoters may also influence the expression of non-coding RNAs, such as long non-coding RNAs (lncRNAs) and microRNAs (miRNAs), which have emerged as key regulators of gene expression in a variety of cancers, including gynecological malignancies.

## 1. Introduction

Complex transcriptional programs are largely regulated through non-coding DNA sequences called cis-regulatory elements (CREs), which activate or repress gene expression in response to different cellular stimuli. CREs are typically non-coding segments of DNA distributed in the vicinity of their target genes, ranging from core promoters found close to the regulated gene sequence to distant elements including enhancers, super-enhancers, silencers, and insulators [1,2]. CREs contain DNA binding sites for transcription factors (TF) and other transcriptional regulators. Together, these complexes provide a functional topology for the chromatin, consisting of physical contacts between the CREs and their target gene promoters through long-range chromatin interactions [3]. The DNA sequences and functional characteristics of CREs are described below.

### 1.1. Transcription Factor-Binding Sites at Gene Promoters

A promoter is a DNA region between 20 and 1000 bp upstream of the transcription start site TSS, which has particular binding sites for transcriptional factors involved in gene transcription. Promoters serve as platforms for recruitment of the transcriptional machinery and pre-initiation complex (PIC) assembly [4,5,6].

### 1.2. Enhancer Elements

**Figure 1:**
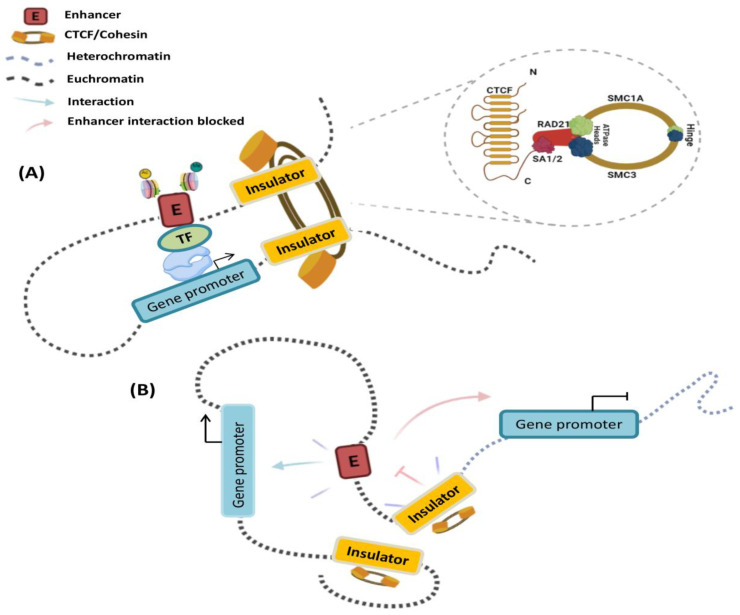
Cis-regulatory elements (CREs) in gene promoters activate or repress gene expression in different cellular contexts: (**A**) A model of transcriptional activation by chromatin looping and enhancers is shown. The CTCF—cohesin interaction promotes the creation of chromatin loops. In somatic cells, this is organized into four core sub-units: structural maintenance of chromosomes protein 1A (SMC1A), SMC3, double-strand-break repair protein rad21 homologue (RAD21), and either cohesin component SA-1 (STAG1) or SA-2 (STAG2). Transcription factors (TFs) bind specifically to sequences of cis-regulatory elements and initiate transcription. (**B**) An insulator can block the spread of heterochromatin and can selectively protect enhancer—promoter interactions inside the chromatin loop. Furthermore, an enhancer-blocking insulator complex restricts the activity of a distal enhancer in an orientation-dependent way.

These elements have a broadly similar structure and function to promoters. The primary difference is their location, as they can be found up to 1 Mb upstream or downstream from their target gene, or even within introns of nearby genes [7,8]. Enhancer elements serve as docking platforms enriched in specific TF binding sites, where the binding of pioneering TF can recruit additional co-activator proteins, including the histone deacetylases p300/CBP, large multi-protein complexes such as Mediator, or even cell type- and lineage-specific co-activators crucial to determining cell fate (Figure 1) [9,10,11,12].

The Mediator complex can mediate chromatin loops by interacting with other TFs and long non-coding RNAs. This interaction facilitates close contact between the enhancer and the promoter, as well as recruitment of the RNA polymerase II (RNA pol II) [13,14]. Thus, in simplified terms, enhancer regulation of target genes occurs in four steps: (i) TF binding to DNA consensus sequences; (ii) coactivator recruitment; (iii) physical interaction with target gene promoters; and (iv) stimulation of the transcription elongation step. Furthermore, depending on their functionality, enhancers can be sub-divided into neutral/intermediate state, which are generally enriched in mono-methylation of lysine 4 of histone H3 (H3K4me1), or active state, labeled by the acetylation of lysine 27 of histone H3 (H3K27ac) [15,16]. These histone post-translational modifications (PTMs) are catalyzed by MLL3/4/COMPASS and p300/CBP, respectively [17,18].

### 1.3. Super-Enhancers and Silencers 

Other important CREs are the super-enhancers, described as clusters of enhancers [19]. The most important characteristics of super-enhancers are that they present higher enrichment of H3K27ac, H3K4me1, Mediator complex (MED1), BRD4, and cell-type specific TFs, compared with normal enhancers [20,21]. Super-enhancer-associated genes have been shown to be physiologically involved in defining the identities of various cell and tissue types. For example, super-enhancers linked with oncogenic genes such as c-MYC have been found in 18 human cancer cells, but not in their healthy counterparts [22]. Additionally, in pluripotent embryonic stem cells (ESCs), super-enhancers contain high levels of master transcription factors such as Oct4, Sox2, Nanog, Klf4, and Esrrb, suggesting that super-enhancer-associated genes might have an impact on cell identity [23].

On the other hand, silencer elements are binding sites for a set of transcription factors known as repressors, which silence the transcription of their target genes; however, silencers have features similar to enhancers, as their function is independent of the orientation and distance to the promoter, and their repressor function appears to operate by blocking the binding of a nearby activator or by directly competing for the same site [2]. Alternatively, a repressor may block transcription by inhibiting PIC assembly [2,24].

### 1.4. Insulator Elements 

In contrast to enhancers and promoters, insulators do not directly regulate gene expression; however, they have enhancer blocking or chromatin barrier functions, both of which depend on the binding of proteins, such as the CCCTC binding factor (CTCF), to form DNA-CTCF complexes (Figure 1). CTCF is a protein with 11 zinc finger domains that binds to the non-palindromic consensus sequence CCGCGNGGNGGCAG [25]. It was initially found as a transcriptional regulator of the chicken c-myc gene, but it is now recognized as a multivalent protein with several activities in genome organization. Enhancer blocking by DNA-CTCF complexes prevents communication between adjacent regulatory elements in a position-dependent manner; for instance, insulators can prevent the activation of a promoter by an enhancer when placed between them (Figure 1). Insulators create a boundary in the chromatin that prevents the spread of heterochromatin [26,27]. Therefore, these regulatory landscapes organize the physical interactions between CREs and their target promoters to coordinate temporal and spatial gene expression. These observations raise the question of how the genome is organized in three-dimensional (3D) space to facilitate specific long-range interactions, while avoiding detrimental ones.

## 2. The Interplay of Cis-Regulatory Elements Is Framed into a 3D Chromatin Structure

**Figure 2:**
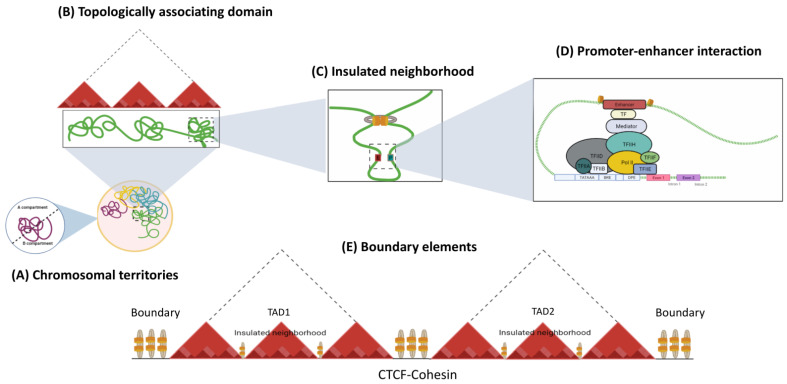
Hierarchical organization of genome. The nucleus in mammalian cells is structured into chromosomes, which have a non-random distribution. (**A**) In interphase, each chromosome is located into discrete sub-nuclear domains called chromosomal territories (CT). Within interphase chromosomes, chromatin folds into (**B**) TADs, which are areas where frequent interactions occur between specific CREs and genes at the local level. (**C**) Insulated neighborhoods are loops constructed by CTCF/cohesin-bound anchors harboring genes and CREs that control gene expression. (**D**) The interaction between CTCF and cohesin facilitates the formation of chromatin loops, where Transcription factors (TF), such as pioneering TFs, bind to enhancers, allowing for the recruitment of the Mediator complex, which further assembles basic transcription machineries at the gene promoter and activates transcription. (**E**) The regions bordering TADs or TAD boundaries regulate gene expression by restricting interactions between adjacent CREs from distinct TADs, avoiding incorrect interactions.

Genome function is a dynamic and flexible concept by nature, as each cell type requires the coordinated expression of genes that contribute to its fate and physiological properties. The three-dimensional organization of the genome is critical for cell identity, as it constantly evolves during adaptation to the environment [14,28,29]. Interphase nuclei show a complex and dynamic architecture of chromosomes and nuclear features. Chromosomes are structured inside the nuclear volume and occupy different regions, called chromosomal territories [30], which correspond to the highest level of hierarchical organization. Within Chromosome Territories (TCs), chromosomes are considered to be separated into two compartments. The A compartments comprise the internal regions of the nucleus, with genes that are usually actively transcribed, whereas the heterochromatic B compartments occupy the periphery of the nuclei and contain inactive genes [31]. Within a chromosomal territory, DNA loops are formed, which fold to build higher-order 3D structures known as topology-associated domains (TADs; see Figure 2) that are enriched and defined by insulator borders associated with CTCF [32]. Finally, inside each TAD, chromatin connections are fostered between a promoter region and enhancer, contributing to the shape transcription by limiting physical contact between regulatory elements [33,34].

Regarding the insulating elements, a study has identified that CTCF and the cohesin complex of structural proteins are the main players in the formation of chromatin loops. These proteins also contribute to the insulator function constraining the heterochromatin-associated position-effect variegation (PEV) phenomenon and mediate a large part of intra-chromosomal interactions [33,35,36]. CTCF-binding sites are enriched in the boundaries between TADs, as well as within intra-TAD chromatin loops (Figure 2) [34,37]. Cohesin, on the other hand, is a ring-shaped protein complex composed of SMC1A, SMC3, RAD21, and either SA1 or SA2 [38,39,40]. Evidence has suggested that the Mediator helps to initiate enhancer—promoter contact, followed by the recruitment of cohesin-loading proteins: the NIPBL/MAU2 complex [41,42]. CTCF and cohesin are thought to mediate TAD and loop formation by an extrusion model; in which, once cohesin is loaded into chromatin, its translocation forms a nascent loop until convergently oriented CTCF proteins are found [41,43]. The cohesin protein component SMC plays a key role in loop extrusion. SMC complexes form enormous rings that are believed to wrap DNA strands. The movement of the SMC ring through the DNA is due to motor activity controlled by one or both ATPase domains of the SMC protein sub-unit, which permits unfettered sliding throughout the DNA [41,44]. This results in a dynamic picture of loops that is constantly developing [45].

Mechanistically, CREs regulate their target genes by physically associating with their promoters through chromatin looping to form these long-range physical interactions. Although CTCF and cohesin have doubtlessly been shown to be essential for chromatin looping, other structural proteins are also involved (Figure 2). For example, the Mediator complex and Yin Yang 1 (YY1), which interact with cohesin and CTCF, respectively, have been proposed to mediate intrachromosomal contacts in interphase cells [14,46,47]. Nevertheless, evidence has suggested that the mechanism by which CREs find the appropriate gene target depends heavily on the TADs structure and CTCF boundaries.

## 3. A Disrupted Landscape of Topologically Associating Domains in Breast and Gynecological Malignancies

Transcriptional dysregulation of cancer-related genes and oncogenic non-coding RNA produces alterations in cell identity, indicating that the 3D chromatin architecture has a central function in governing gene transcription, cancer development, and cellular heterogeneity. In cancer cells, disruption of TAD structure or inter-TAD changes may cause chromatin rewiring, leading to the overexpression of oncogenes or down-regulation of tumor suppressors [48,49].

Structural TAD alterations or inter-TAD modifications occur in the cancer genome by different mechanisms, including alteration to CTCF (mutations, aberrant DNA methylation, or post-transcriptional modification) [43,50,51,52], cancer-risk single nucleotide polymorphisms (SNPs), viral integration, hormonal response, and structural variants (SVs), such as deletions, insertions, inversions, duplications, or translocations [53,54].

## 4. CTCF Alterations Disrupt the 3D Structure of Chromatin

**Figure 3:**
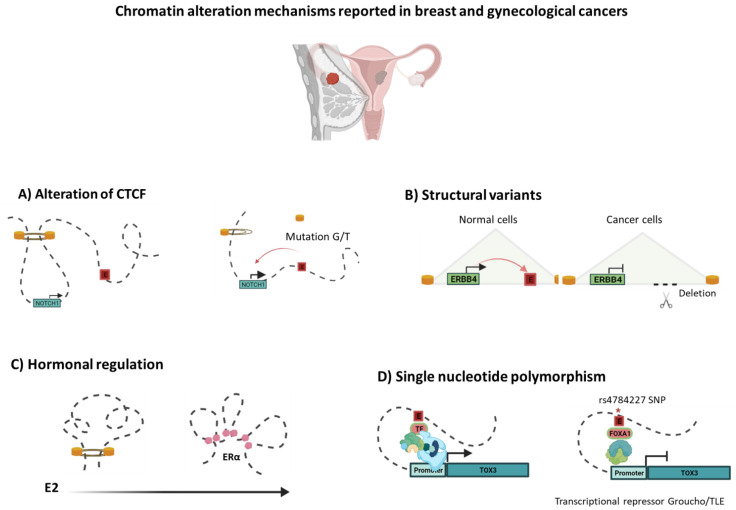
Chromatin alteration mechanisms reported in breast and gynecological cancers. As proper chromatin structure folding is essential for gene regulation, disruption of the TAD structure or inter-TAD modifications may lead to rewiring of chromatin connections to activate certain oncogenes or down-regulation of tumor suppressors. Breast and gynecological cancer genomes have been reported as having diverse alterations, including: (**A**) Mutations in the CTCF motifs anchors (G/T) at the boundary of the TAD motifs, leading to NOTCH1 overexpression. (**B**) Alternatively, structural variants, such as deletions, in cancer genomes can frequently delete enhancers, contributing to oncogenesis by decreasing tumor suppressor gene expression (e.g., ERBB4) [53,54]. (**C**) Another mechanism that has been linked to dynamic changes in chromatin structure is hormonal response. Constant E2 stimulation promotes 3D genome re-compartmentalization by ERa binding, generating chromatin interactions of invasion-related oncogenes [59]. (**D**) Single nucleotide polymorphisms (SNPs) often exist in CREs, which may cause changes in interactions. The rs4784227 SNP alters the sequence of recognition of an enhancer that controls TOX3 gene transcription, favoring the binding of the FOXA1 transcription factor and Groucho/TLE, resulting in decreased expression of the TOX3 tumor suppressor gene [60].

Breast and gynecological cancers, such as ovarian cancer, share common genetic and non-genetic risk factors including mutations in BRCA1 and BRCA2, the most significant risk factors for both cancers, suggesting that similar biological mechanisms drive breast and ovarian cancer development. Mutations in the CTCF gene have been reported in breast cancer, endometrial cancer, and ovarian cancer [55,56]. These mutations are predominantly mis-sense or nonsense and, thus, have been predicted to impair CTCF function [55]. Some tumor cell mutations occur within the zinc fingers of CTCF and may selectively perturb certain loops, as they affect CTCF binding at only a subset of sites [57]. For example, in ovarian cancer, mutations in the CTCF motif anchors (G/T) at the boundary of the TAD motifs lead to NOTCH1 overexpression, most likely through inappropriate enhancer action caused by TAD disruption (Figure 3). Deregulation of the NOTCH signaling cascade has been linked to embryonic development, cell proliferation, and growth in many types of cancer [58].

Interestingly, viral integration can drive oncogenesis by chromatin reorganization. Mehran et al. have shown that the viral integration of human papillomavirus (HPV) introduces new CTCF binding sites in the cervical cancer genome.

This promotes local changes in the expression of genes related to tumor viability, such as FOXA, KLF12, SOX2, CUL2, CD274, and PBX1, and the viral oncogenes E6 and E7 that mediate mitogenic and anti-apoptotic stimuli, by interacting with numerous regulatory proteins of the host cell that control the cell cycle in some HPV+ tumors [61].

In cancer cells, CTCF can also undergo a number of post-translational modifications which change its properties and functions. One such modification which has been linked to cancer is poly (ADP)-ribosylation (PARylation) at the n-terminal domains of CTCF, which promote the insulator and transcription factor functions of CTCF, while phosphorylation impairs its DNA binding activity [62]. CTCF phosphorylation at threonine (T) 374 and serine (S) 402 has been observed in breast cancer [63]. The Hippo-LATS signaling pathway is a key regulator of cell proliferation, apoptosis, tissue homeostasis, and tumorigenesis. LATS1/2 kinases are the key players in this cascade, which phosphorylate the YAP protein and cause its sequestration in the cytoplasm, resulting in cell apoptosis and growth arrest. In breast cancer, YAP is overexpressed and functions as a transcriptional coactivator for a group of genes that facilitate cell growth and survival. In MCF7 cells, it has recently been discovered that LATS kinases phosphorylate CTCF at one of its zinc fingers, impairing its DNA binding and canceling the chromatin looping of YAP target genes (AMOTL2, AXL, CRY1, GLI2), suggesting that CTCF-mediated insulated neighborhoods could be necessary for YAP target gene activation [63].

Finally, the aberrant transcriptional silencing of tumor suppressor genes is accompanied by dynamic changes in chromatin structure mediated by CTCF. For instance, CTCF plays a role in establishing and maintaining the tumor suppressor p16 in higher-order chromosomal domains through appropriate boundary formation. p16 is a key regulator of cell cycle arrest in G1 and senescence, primarily through inhibition of the cyclin-dependent kinases CDK4 and CDK6. In fact, inactivation of the p16 gene by promoter methylation is one of the earliest losses of tumor suppressor function in numerous types of human cancers, including breast cancer. In p16-expressing MDA-MB-435 breast-cancer cells, CTCF binds downstream of the region enriched for heterochromatin marks located within −2 kb and +1 kb of the active promoter of the p16 gene; however, in T47D breast cancer cells that contain aberrantly silenced p16, the boundary domain at −2 kb disappears and prevents CTCF binding, hereby promoting the spread of heterochromatin marks throughout the entire p16 promoter region. These data raise the possibility that the dissociation of CTCF from p16 during early tumorigenesis is not due to DNA methylation alone but may result from loss of function of CTCF to organize a boundary or insulator element. This, in turn, would result in secondary changes in chromatin structure that are incompatible with CTCF binding to DNA [64].

## 5. Hormones Drive Dynamic Transitions in Chromatin Architecture Which Influence Gene Expression

Hormones, which are known to have strong effects on gene function, may induce dynamic changes in chromatin organization. In the 3D genome, estrogen therapy has been shown to induce enhancer-promoter interactions through chromatin looping structures around target genes, including the keratin gene cluster, NR2F2, and SIAH2, as well as organizing the suppression of genes down-regulated in breast cancer basal-like sub-types, such as TAOK2 [65,66]. Surprisingly, TADs function as units of steroid hormone response. Some TADs contain binding sequences for the estrogen receptor (ESR1) and progesterone receptor (PGR), which are referred to as hormone-control areas (HCRs). In T47D cells, Dily et al. discovered over 200 HCRs with ESR1 and PGR binding sites that form a looping pattern, allowing intra-TAD interactions and enhancing the transcriptional activity of genes located in TADs with HCRs.

One of these TADs, for example, includes the ESR1 gene as well as five other protein-coding genes, including ZBTB2, RMND1, ARMT1, CCDC170, and SYNE1, all of which are coordinately controlled by estradiol (E2) and progestins (Pg). In the absence of hormones, ESR1-TAD maintains a basal expression of resident genes. In comparison, when exposed to Pg and E2, ESR1-TAD is restructured to create intra-TAD interactions and appears to increase transcriptional activity [67]. Similarly, E2 and the synthetic progestin R5020 have been shown to promote the binding of PR and the transcriptional factor Paired box gene 2 (PAX2) in Ishikawa endometrial cancer cells, resulting in enhancer-promoter interacting loops within a TAD with HCRs, which contains several tumor development genes such as HMGA2, ETV4, ETV7, and GZMB [68].

Additionally, Zhou et al. have demonstrated a complex 3D chromatin structure over a time course of E2 stimulation in breast cancer cells MCF7 and T47D. Constitutive estrogen stimulation increased ERa binding but decreased CTCF binding. ERa promoted the re-compartmentalization of interactions in TADs enriched with genes related to invasion, aggressiveness, or metabolism signaling pathways; ECM receptor interaction; focal adhesion; and the cell cycle (Figure 3) [59].

## 6. TAD Organization Can Be Rewritten by Structural Variants

TAD organization may be influenced by structural variants, potentially leading to gene expression changes and disease. Deletions affecting TAD boundaries may trigger gene dysregulation by TAD fusion, while duplications may form neo-TADs, the pathogenicity of which is determined by the regulatory elements and genes contained inside. Furthermore, inversions involving TAD boundaries may change the relative position of regulatory elements, causing changes in phenotypes through novel enhancer—promoter interactions [69].

Translocations of large portions of chromosome arms have a potential impact on gene expression by TAD alteration [54]; for example, Zinc Finger Protein 703 (ZNF703) is a typical Luminal B breast cancer oncogene that is found on chromosome 8. ZNF703 has been linked to the activation of the Akt/mTOR signaling pathway, as well as the activation of pluripotency-related genes [70,71]. ZNF703 overexpression has been attributed to chr8-chr14 translocation in 7 out of 10 breast cancer samples and T47D breast cancer cells. This translocation joins the chr8: ZNF703/FGFR1 to the chr11: CCND1 and seems to generate a neo-TAD near the ZNF703, promoting gene overexpression [72].

SVs, such as oncogene amplification, have been shown to promote tumorigenesis by altering the number of copies of entire genes [52]. Additionally, it may cause disease by disrupting TADs or the fusion of adjacent to TADs, enabling enhancers from neighboring TADs to trigger oncogenes and resulting in gene dysregulation [73,74]. FOXA1 overexpression comprises a major proliferation and survival signal for luminal type A breast cancer, which has been attributed to the amplification of FOXA1 in around 6% of primary tumors and 10% of metastatic ER+ (estrogen receptor positive) tumors [75,76]. In breast tamoxifen-resistant (TamR) breast cancer cells, Fu et al. observed that FOXA1 amplification rewrites the 3D chromatin structure to promote super-enhancer (SE) acquisition with FOXA1 binding sites. Furthermore, these SE elements are enriched in specific TF-binding sites, such as AP-1, STAT5, SOX9, and SMAD3, which are associated with stemness, epithelial—mesenchymal transition, and aggressive tumor behavior [77]. Similarly, overexpression of the Desmoglein 3 (DSG3) oncogene has been correlated with DSG3 gene amplification in breast cancer tissues. DSG3 gene amplification occurs at the TAD border, resulting in overexpression [78], which is associated with cancer cell proliferation and invasion through a plakoglobin-mediated signaling pathway [79].

In parallel, analysis of whole-genome sequencing data in endometrial cancer showed an amplification encompassing a 10 kbp non-coding area harboring a super-enhancer, as identified by the H3K27ac ChIP-seq profile, located ~800 Kbp downstream of the MYC gene transcription start site. Chromatin conformation capture (3C) assays in Ishikawa cells have revealed a physical interaction between super-enhancers and the MYC promoter. Furthermore, tumors with amplifications near the MYC locus have higher MYC expression than tumors without amplification [80]. Alternatively, deletions in cancer genomes can frequently delete enhancers, contributing to oncogenesis by decreasing tumor suppressor gene expression. In breast cancer, ERBB4 acts as a tumor suppressor, regulating cell proliferation, development, and differentiation. Nonetheless, as compared to the enhancer’s annotations in HMEC cells, a set of enhancers found upstream of ERBB4 was found to be deleted in T47D breast cancer cells. Previous studies have demonstrated that several enhancers interact with the ERBB4 promoter, implying that the elimination of this area can have an impact on ERBB4 expression, thereby contributing to breast cancer (Figure 3). Surprisingly, T47D’s deleted enhancers are located close to genes involved in the cellular reaction to VEGF, genes down-regulated in breast cancer, and genes involved in DNA repair abnormalities [81]. KLF5 has been linked to oncogenic characteristics in previous research. Overexpression of KLF5 has been shown to enhance carcinogenesis in cervical cancer patients, in addition to its role as a positive regulator of cancer cell proliferation [82,83]. Furthermore, overexpression is a predictive marker for poor prognosis in breast cancer patients [84].

KLF5 up-regulation is assisted by focused amplification of super-enhancers located 600 kb apart between KLF5 and KLF12. The amplified super-enhancers are found inside the same TAD as the promoter region and gene body of KLF5, but not the promoter or whole gene body of KLF12, indicating that KLF5 is the candidate target gene, according to Hi-C data [85].

## 7. Cancer-Risk Single Nucleotide Polymorphisms Promote Pathogenic Promoter-Enhancer Interactions

Single nucleotide polymorphisms (SNPs) often exist in CREs, which may result in pathogenic promoter—enhancer interactions and, therefore, aberrant gene expression, according to genome-wide association studies (GWAS). SNPs in CREs may alter the DNA recognition motifs of TFs. These alterations cause them to interact with other elements differently, influencing changes in intra-TAD interactions and gene expression [49]. Indeed, the variant risk allele of the rs4784227 SNP alters the sequence of a Forkhead DNA recognition motif inside an enhancer that controls TOX3 gene expression, favoring the binding of the FOXA1 (Figure 3). The increased binding of FOXA1 represses the enhancer’s transactivation capacity through recruitment of the transcriptional repressor Groucho/TLE, resulting in decreased expression of the TOX3 tumor suppressor gene, providing additional proof of genetic alterations targeting enhancers in cancer [60].

In endometrial cancer, Painter et al. have described a putative regulatory element that interacts with AKT1—a member of the PI3K/AKT/MTOR intracellular signaling pathway—and negatively affects AKT1 expression. Association and functional analyses have demonstrated that the SNP rs2494737 maps to this silencer element, affecting its regulatory capability over the AKT1 promoter, hence resulting in increased AKT1 expression in both Ishikawa and EN-1078D endometrial cancer cells [86]. Cyclin D1 is one of the most significant cell cycle regulators. It is a member of the D-type cyclin family, which regulates cell cycle progression from G1 to S phase by modulating the function of cyclin-dependent kinases (CDKs). In addition, CCND1 is also a well-known oncogene that plays a key role in cell cycle progression and whose overexpression in breast cancer has been linked to poor prognosis. The CCND1 promoter is located 125 kb downstream of a putative regulatory element 1 (PRE1 enhancer), and regularly interacts in ER+ MCF7 and T47D cells. Surprisingly, breast cancer SNPs (rs78540526 and rs554219) in PRE1 almost completely abolished enhancer function and decreased the amount of cyclin D1 protein in MCF7 cells [87]. This evidence demonstrates that disease-linked regulatory SNPs can impact chromatin 3D interactions between genes and regulatory elements to modulate target gene expression.

A recent GWAS study revealed that the SNPs rs1011970 and rs615552 increase the risk of breast and gynecological malignancies in a region on chromosome 9p21 [88,89].

9p21 comprises three genes: CDKN2B (encoding p15ink4b), CDKN2A (encoding p16INK4a and p14ARF), and the 3′ end of CDKN2BAS (an anti-sense non-coding RNA in the INK4 locus; ANRIL) [90]. Farooq et al. have previously found an enhancer cluster adjacent to the INK4a gene with 24 core enhancers (E1–E24) in HPV-positive cervical tumors. This enhancer cluster is closely linked to the transcriptional activation of CDKN2A, which physically interacts with five enhancers, according to a chromosomal conformation capture approach (4C): E5, E8, E12, E17, and E19. Individual deletion of the three interacting enhancers (E8, E12, and E17) suppressed INK4a, ARF, and INK4b promoter transcription, and HPV-positive cell proliferation and migration were hampered when a single enhancer is deleted [91].

## 8. Chromatin 3D Alterations: miRNAs and lncRNAs Landscapes

CREs also play a direct role in non-coding RNA (ncRNA) expression regulation, through long-range chromatin interactions. Genes encoding microRNAs (miRNAs) and long non-coding RNAs (lncRNAs) are transcriptionally regulated in a way similar to protein-coding genes. The first confirmation of this emerged from early biological experiments, which showed many cases of miRNAs and lncRNAs being transcribed by RNAPII [92,93]. Additionally, there is evidence that a set of RNAPII-associated transcription factors, such as c-Myc, cAMP-response element binding protein (CREB), SP1, p53, and MyoD [94,95,96], regulate miRNA and lncRNA expression. Notably, for some ncRNAs associated with cancer, 3D chromatin interactions could be rewritten, leading to enhancer—miRNAs- or enhancer—lncRNAs-altered interactions promoting changes in ncRNA expression [97].

## 9. Enhancer-miRNAs Interactions in Gynecological Cancers

MicroRNAs (miRNAs) are single-stranded RNAs of 22 nucleotides that post-transcriptionally negatively regulate the expression of their target genes, through targeting specific sites in the 3′ untranslated region (3′ UTR) of mRNA [98]. A great deal of studies in the literature have confirmed that miRNAs are deregulated in almost all known cancers, including breast and gynecological cancers [99,100].

Additionally, miRNA expression is involved in the balance of oncogenes and tumor suppressor genes. Let-7 miRNA, for example, has been found to be down-regulated in a range of cancers, including gynecological and breast cancer. Let-7 is a significant negative regulator of oncogenes, such as RAS, HMGA [101], c-Myc [102], and ERa [103], according to in vitro investigations. Let-7 overexpression, for example, causes ERa signaling pathway suppression, reduces cell proliferation, and promotes cell death in breast cancer [103]. Let-7 also inhibits cell growth in cervical cancer cells by down-regulating RAS, suggesting that this miRNA may act as a tumor suppressor [104].

On the other hand, miR-21 was one of the first miRNAs to be linked to cancer, having been found to be abnormally overexpressed in a variety of malignancies. Cell proliferation, migration, and invasion were significantly reduced when miR-21 was knocked down. miR-21 has been found to target many tumor suppressor genes in breast and ovarian cancer, including BCL2, PDCD4, and PTEN [105,106,107]. These studies have indicated that miRNAs play an essential role in post-transcriptional processing and gene silencing in cancer cells, by inhibiting protein translation or degrading polypeptides by binding complementarily to the 3′ UTR of target mRNAs.

However, research on miRNA transcriptional regulation is an emerging field that aims to explain the aberrant miRNA expression in cancer through the alteration of chromatin interactions, showing that enhancers regulate not only protein-coding genes but also miRNAs. A total of 2418 enhancer—miRNA associations have recently been discovered in 31 human cancers, including invasive breast carcinoma and ovarian serous cystadenocarcinoma. The target genes of miRNAs regulated by enhancers were found to be substantially involved in the P53, PI3K/AKT/mTOR, and MAPK signaling pathways, as well as stem cell, focal adhesion, and cell cycle and chromatin organization processes [108]. Some cellular processes, such as epithelial-mesenchymal transition (EMT), which is TGF-β1-mediated, are thought to be initiated by chromatin reorganization events. A major player in this process is the miR-200 gene family, which comprises five members: miR-200a, miR-200b, miR-200c, miR-141 and miR-429. These are required for epithelial state maintenance, while remaining silent in mesenchymal cells, leading to increased cell motility, proliferation, and migration. One of the mechanisms that controls miR-200 family transcriptional activity is an enhancer element located roughly 5.1 kb upstream of miR-200a, miR-200b, and miR-429. The minimal promoter region stimulated their transcription approximately 27-fold in breast epithelial cells, but had little or no activity in mammary mesenchymal cells, suggesting that this enhancer element is important for miR-200b/-200a/-429 expression in breast epithelial cells [109].

On the other hand, the interaction of super-enhancers (SEs) with miRNAs has recently been identified by Suzuki et al., who revealed changes in the loss or gain of SEs around miRNA genes in breast cancer. miRNAs with SE gain (miR-21, miR-17, and miR-19b) were associated with oncogenic functions, and SE loss was correlated with tumor-suppressive miRNAs (let-7b, miR-145, and miR-193b), which partly explains the increased or decreased expression reported for these miRNAs in breast cancer tissues and the endometrium [110].

Another example is the miR-196a expression landscape. This miRNA has been found to be overexpressed in ovarian and breast cancer [111,112]. miR-196b modulates cancer cell proliferation by inhibiting CDKN1B, promoting cancer cell migration and invasion [111]. Increased miR-196a expression has been linked to a loss of DNA methylation in the miRNA’s promoter regions, as well as long-range transcriptional regulation with the HOTAIR enhancer (HOTAIR distal enhancer, HDE) and a new miR-196a-Enhancer. 3C assays and luciferase reporters have clearly shown that both enhancer elements interact physically with each of the miR-196a promoter regions [112]. However, the SNP rs11614913 within the miR-196a gene has been associated with a lower risk of breast cancer and decreased miR-196a expression [112,113,114]. This SNP may have a negative effect on the interaction of enhancers with the miR-196a promoter.

## 10. Enhancer-lncRNAs Interactions in Gynecological Cancers

A large number of lncRNA genes are encoded in the human genome [115], and have been identified as key regulators of transcriptional networks during cancer progression.

The size threshold most typically used for operationally defining lncRNAs is 200 nt [116], with either no or very short ORFs. To some extent, lncRNAs are similar to mRNAs, as they are often 5’-capped, polyadenylated, spliced, and transcribed by RNA pol II [116,117]. lncRNAs can function as molecular signals, decoys, RNA guides, miRNA sponges, or scaffolds to control gene expression (for further reading, see [118,119,120]) and, as a result, they can operate as oncogenes and tumor suppressors. However, abnormal lncRNA expression can be a determining factor in the cancer etiology, promoting tumor development and disease progression, as well as driving metastasis and therapy resistance.

HOTAIR, for example, is one of the most-researched oncogenic lncRNAs, the overexpression of which has been found to be up-regulated in both primary and metastatic breast and ovarian malignancies. Its overexpression correlates with increased cell proliferation and metastasis [121,122]. HOTAIR potentially binds to the repressive polycomb 2 complex (PRC2) to negatively regulate the expression of P53 and p21 in MCF-7 and MDA-MB-231, while down-regulation of this lncRNA leads to the activation of p21 and cell cycle arrest at G1 phase [123].

MALAT-1 is another lncRNA which has been shown to be overexpressed in breast and gynecological cancers [124,125]. Specifically, in the cervical cancer cell line CaSki, siRNA-mediated down-regulation reduces proliferation and migration [126]. MALAT-1 inhibits apoptosis in cervical cancer by up-regulating the anti-apoptosis genes Bcl-2 and Bcl-XL, while inhibiting the apoptosis genes caspase-3 and caspase-8 [126]. Similarly, data has suggested that the lncRNA RNA H19 is overexpressed in breast, cervical, and ovarian cancers [127,128,129]. In ovarian cancer, H19 overexpression has been observed in 12 of 16 patients and ovarian cancer cell lines, including OVCAR-3, SKOV-3, and OV-90. H19 ectopic expression enhanced cell proliferation, whereas H19 siRNA treatment activated apoptosis [127].

These results demonstrate that lncRNAs are key regulators of gene expression, and their research in the context of cancer has mainly been focused on the mechanisms by which they control gene expression; however, the processes involved in their own expression—particularly through 3D chromatin interactions as regulators of lncRNA expression—remain poorly understood. The first lncRNA gene, found in Burkitt’s lymphoma translocations, was Plasmacytoma variant translocation 1 (PVT1) [130]. The PVT1 gene, according to previous research, encodes an oncogenic long non-coding RNA (lncRNA) that aids breast cancer cell growth in vivo. Many SVs may be observed around the PVT1 promoter region of breast cancer cells, including deletions, inversions, and duplications [131,132]. Recently, Cho et al. have proposed alternate tumor suppressor functions for the PVT1 promoter, independent of PVT1 lncRNA. In this model, PVT1 and MYC oncogene promoters, positioned 55 kilobases apart on chromosome 8q24, compete for interaction with four intragenic enhancers in the PVT1 locus, enabling the PVT1 promoter to restrict MYC oncogene expression. However, deletion of the PVT1 transcription start site reduced interactions between these four enhancers and the PVT1 promoter, hence enabling interactions with the MYC promoter and, thus, increasing its expression in the MDA-MB-231 cell line. Finally, the presence of SVs in breast cancer samples impairs the PVT1 promoter’s tumor suppressor action [132].

**Figure 4:**
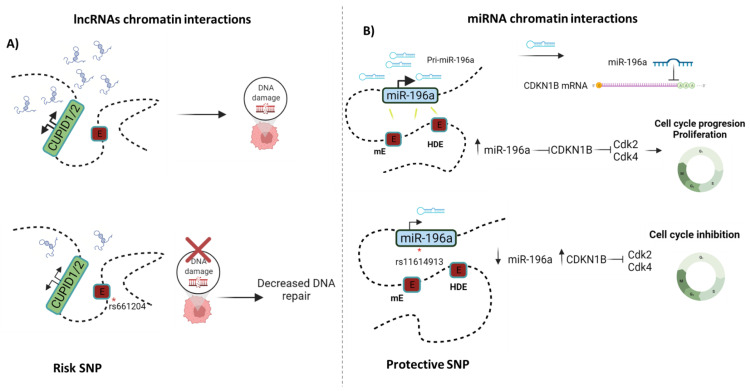
ncRNA-chromatin interactions: (**A**) Enhancer-lncRNA interactions. CUPID1 and CUPID2 are two lncRNAs that are transcribed from a bi-directional promoter and regulated by the enhancer PRE1. When DNA is damaged, CUPID1/CUPID2 favor double-strand break repair. However, reduced chromatin looping between the enhancer and the bi-directional promoters of CUPID1 and CUPID2 has been reported as being mechanistically responsible for the breast cancer risk-associated SNPs rs661204 [133]; (**B**) Enhancer-miRNA interactions. In breast cancer cells, increased miR-196a expression is associated with HOTAIR-enhancer and miR-196a-enhancer. Furthermore, overexpression of this miRNA is linked to proliferation through suppressing CDKN1B. A protective SNP rs11614913 within the miR-196a gene has been linked to a lower risk of breast cancer and decreased miR-196a expression, suggesting a weaker interaction between enhancers with the miR-196a promoter [112].

The PRE1 enhancer is an example of an alteration in chromatin interactions caused by SNPs. PRE1 interacts with the CUPID1/2 lncRNA promoters. Subsequent activation of the CUPID promoter by PRE1 induces the expression of CUPID1 and CUPID2, which are required for the DNA damage response, increasing their expression in ER+ cancer cells, as has been determined in MCF7 and T47D cells [82]. However, the presence of risk-SNPs (rs661204 and rs78540526) reduces both the activity of PRE1 and chromatin looping between PRE1 and the CUPID promoter (Figure 4). In contrast, in CAL51 cells—a breast cancer cell line heterozygous for the risk-SNPs—it has been demonstrated that chromatin looping between PRE1 and the CUPID1 and CUPID2 promoters was abrogated. These findings indicate that alterations in chromatin interactions caused by SNPs can impact lncRNA expression, resulting in lower CUPID1 and CUPID2 expression. The loss of CUPID1 and CUPID2 expression causes pRPA and RAD51 recruitment to be altered, resulting in decreased DNA repair [133].

Interestingly, some studies have demonstrated that abnormal interactions among lncRNAs and super-enhancers occur in cancer cells. For example, DSCAM-AS1 is a cancer-related lncRNA with high expression in Luminal A, B, and HER2-positive breast carcinoma, where its expression drives breast cancer proliferation. A set of super-enhancers has been discovered adjacent to the TSS of DSCAM-AS1, by analyzing the enrichment of H3K27ac in MCF-7 cells. Through 3C-based techniques, it was uncovered that the SE and DSCAM-AS1 TSS engage in long-range chromatin oncogenic interactions to promote the transcription of this lncRNA in breast cancer [134]. Furthermore, the anti-sense lncRNA HOXA Transcript Antisense RNA, Myeloid-Specific 1 (HOTAIRM1), has been found to be highly expressed in breast and endometrial cancers [135,136]. Previous research has shown that HOTAIRM1 interacts directly with EZH2 and inhibits the PRC2 complex from binding and depositing H3K27me3 to induce HOXA1 oncogene overexpression. Interestingly, HOTAIRM1 overexpression has been associated with an enhancer situated 150 kb downstream of HOXA1 [137], and previous research has identified HOTAIRM1 as a major regulator of proliferation, migration, invasion, and epithelial—mesenchymal transition (EMT) in vitro [135,138].

In breast cancer, Joshua et al. have shown that the MALAT1 genomic locus contains potential distal enhancers upstream and downstream of the MALAT1 gene body. Importantly, HIF enhances the connection between the MALAT1 promoter and the downstream enhancer in hypoxia; notably, this is only seen in breast cancer cell lines, and not in non-tumorigenic mammary cell lines [139].

Furthermore, Milevskiy et al. have demonstrated a pathological interaction between an enhancer located 150 kb downstream of the HOTAIR TSS and the lncRNA HOTAIR promoter in breast cancer cell lines MCF7, ZR-751, and MDA-MB-453. Additionally, using luciferase reporter assays, it was demonstrated that this enhancer induces a five-fold increase in luciferase expression, as compared to the promoter alone from HOTAIR and other genes, such as HOXC11 [114]. Finally, the lncRNA MIR31HG has been shown to be up-regulated in breast cancer [140] and, through 3C-based techniques, it was demonstrated that an enhancer located 100 kb upstream of the promoter of the intergenic MIR31HG facilitated its overexpression. Interestingly, MIR31HG overexpression has been linked to the activation of genes involved in senescence, such as p16 and p53 [141].

According to these findings, aberrant interactions between enhancer-lncRNAs may enable their mis-regulation, therefore promoting the emergence and advancement of cancer hallmarks.

## 11. Concluding Remarks

The significance of genomic geometry has become clearer in a variety of biological settings. As structural components of chromosomes, TADs are critical functional elements of genomes, serving as regulatory environments for the genes they house. As a result, consideration of genome folding is expected to become more essential in cancer genomic research, and potentially may be considered for the design of novel therapeutic strategies. According to certain authors, there are two major processes that disturb the architecture of the 3D genome. The first is the removal or mutation of a TAD border, while the second includes genomic rearrangements that break apart TADs and generate new ones without altering TAD boundaries directly. However, some research has indicated that certain mechanisms, such as hormone stimulation or epithelial-mesenchymal transition, can stimulate the development of novel chromatin structures locally. Thus, further studies are required to better understand the function of chromatin interactions in cancer.

Recent studies have demonstrated that many ncRNAs, such as lncRNAs and miRNAs, play important roles in human cancers. Similar to coding genes, ncRNA transcription is determined by a series of proximal and distal regulatory elements. Although knowledge regarding the transcriptional control of protein-coding genes is becoming well-established at a global scale, no equivalent data exist for lncRNA and miRNA genes, which have recently been shown to be as abundant as protein-coding genes in mammalian genomes. The control of ncRNA expression takes on increased significance, as they are significant regulators of gene expression and can promote the expression of key genes for cancer development. Interestingly, the 3D architecture of chromatin might act as a regulator of gene expression regulators. Understanding 3D chromatin modifications in ncRNAs may provide insight into the causes of aberrant expression in cancer and, as a result, new treatment methods might be developed.
